# Non-Linear Mixed-Effects Pharmacokinetic Modeling of the Novel COX-2 Selective Inhibitor Vitacoxib in Cats

**DOI:** 10.3389/fvets.2020.554033

**Published:** 2020-09-24

**Authors:** Jianzhong Wang, Benjamin K. Schneider, Hongzhi Xiao, Jicheng Qiu, Xiaohui Gong, Yeon-Jung Seo, Jing Li, Jonathan P. Mochel, Xingyuan Cao

**Affiliations:** ^1^College of Veterinary Medicine, Shanxi Agricultural University, Taigu, Shanxi, China; ^2^Department of Veterinary Pharmacology and Toxicology, College of Veterinary Medicine, China Agricultural University, Beijing, China; ^3^Biomedical Sciences, SMART Pharmacology at Iowa State University College of Veterinary Medicine, Ames, IA, United States; ^4^Beijing Orbiepharm Co. Ltd., Beijing, China; ^5^Key Laboratory of Detection for Veterinary Drug Residues and Illegal Additives, Ministry of Agriculture and Rural Affairs of the People's Republic of China, Beijing, China

**Keywords:** Nlme, NSAIDs, PK/PD, vitacoxib, cats

## Abstract

The objective of this study was to develop a non-linear mixed-effects (NLME) model to describe the disposition kinetics of vitacoxib in cats following intravenous (I.V) and oral (P.O) (single and multiple) dosing. Data from six consecutive studies with 16 healthy neutered domestic short hair cats were pooled together to build a pharmacokinetic (PK) model using NLME. Population PK parameters were estimated using the stochastic approximation expectation maximization (SAEM) algorithm implemented in Monolix 2019R2. A two-compartment mammillary disposition model with simultaneous zero- and first-order absorption best described the PK of vitacoxib in plasma after oral dosing. The systemic CL of vitacoxib was found to be low (110 ml/h), with a steady-state volume of distribution (VSS) of 3.42 L in cats. Results from the automated covariate search in Monolix 2019R2 showed that bodyweight had a significant effect on the central volume of distribution of vitacoxib. Lastly, using Monte Carlo simulations, we investigated the time course of several dosages of vitacoxib from 0.01 to 8 mg/kg. Using this simulation set, we found a range of reasonable dosages that produce therapeutic plasma concentrations of vitacoxib for 24 h or more in cats.

## Introduction

Coxibs are a subclass of non-steroidal anti-inflammatory drugs (NSAIDs) that selectively inhibit cyclooxygenase (COX) and have been widely used for the treatment of inflammation-related pain and fever in human and veterinary medicine (1). However, few coxibs are registered for the management of post-operative pain and inflammation in cats (2). NSAIDs should be used cautiously in cats due to their limited capacity for hepatic glucuronidation, which is the main pathway for metabolism and excretion of coxibs (3). Additionally, cats are particularly sensitive to the gastrointestinal adverse effects of NSAIDs than are other species (4). As of today, five coxibs—cimicoxib, deracoxib, firocoxib, mavacoxib, and robenacoxib—are now licensed for use in dogs, but only one coxib—robenacoxib—is registered for use in cats in several European countries and the USA (5).

Vitacoxib is a highly selective cyclooxygenase-2 (COX-2) inhibitor (6) registered for use in dogs in China for the treatment of pain resulting from orthopedic and soft tissue surgery, osteoarthrosis, and rheumatoid arthritis (7). The safety profile of vitacoxib was initially established in rats (8–11) and more recently reported in young adult horses (12, 13), rabbits (14), beagle dogs (15) and cats (16).

Non-linear mixed-effects (NLME) models are versatile tools for simultaneously modeling pharmacokinetics (PK) and quantifying inter-individual and intra-individual variability in distribution (15, 17–19). Using NLME models, pharmacometricians have the ability to leverage data from multiple studies, dosing routes, and administration schedules (17, 20). Additionally, NLME models are useful for dose selection and covariate identification in companion animals (18).

After fitting an NLME model and estimating individual variability, pharmacologists can use the NLME approach to produce simulations of dosing regimens not originally tested in the experimental design. These simulations can then be used to derive meaningful estimates of effective and safe dosing schedules. The ability to guide experimental design via simulations is particularly relevant in the case of developing viable dosing regimens of vitacoxib in cats, given the known susceptibility of cats to coxibs.

The objective of this analysis was to leverage PK data generated from six different studies using different routes, doses and feeding schedules to characterize the PK of vitacoxib in cats. The effect of sex, bodyweight, and food intake on vitacoxib PK was further evaluated to determine the need for dosing adjustment based on these covariates.

## Materials and Methods

### Animals

Vitacoxib plasma concentration time-course data from six consecutive studies—for a total of 16 healthy, neutered, domestic short-hair cats (1 to 3 years of age, 2.9 ± 0.78 kg bodyweight)—were pooled together for data analysis. Details for cat allocation, vitacoxib dosing, and sampling schedule (dose and time of blood collection) and feeding status can be found in Table 1. Prior to each study start, cats were acclimated to the research facilities for a period of 1 month. Cats were housed in groups but were kept in separate cages. The cats were fed a standard commercial diet and had *ad libitum* access to water. General health assessments were performed daily during the course of each individual study.

**Table 1:** List of vitacoxib dosing and sampling schedules in cats included for NLME data analysis.

**Study**	**Animal ID**	**Feeding status**	**Route**	**Dose**	**Sampling schedule**
Study 1	ID: 9, 10, 11, 12, 13, 14, 15, 16	12 h fasting overnight	P.O	2 mg/kg	0.33, 0.67, 1, 2, 4, 6, 8, 10, 12, 24, 36, and 48 h
Study 2	ID: 9, 10, 11, 12, 13, 14, 15, 16	12 h fasting overnight	I.V	2 mg/kg	0.08, 0.25, 0.5, 1, 2, 3, 4, 6, 8, 10, 12, 24, 36, and 48 h
Study 3	ID: 9, 10, 11, 12, 13, 14, 15, 16	2 h after feeding	P.O	2 mg/kg	0.33, 0.67, 1, 2, 4, 6, 8, 10, 12, 24, 36, and 48 h
Study 4	ID: 1, 2, 3, 4, 5, 6, 7, 8	12 h fasting overnight	P.O	1 mg/kg	0.33, 0.67, 1, 2, 4, 6, 8, 10, 12, 24, 36, and 48 h
Study 5	ID: 1, 2, 3, 4, 5, 6, 7, 8	12 h fasting overnight	P.O	4 mg/kg	0.33, 0.67, 1, 2, 4, 6, 8, 10, 12, 24, 36, and 48 h
Study 6	ID: 9, 10, 11, 12, 13, 14, 15, 16	12 h fasting overnight	P.O	2 mg/kg for 7 days	0.33, 0.67, 1, 2, 4, 6, 8, 10, 12, and 24 h (day 1 and day 7); 36 and 48 h (day 7); 0 and 5 h (day 2 to day 6)

*Vitacoxib was administered by the oral (P.O) or intravenous (I.V) route in the morning at time 0 (the exact time of dosing varied between studies). Times indicated below are hours post-administration*.

### Experimental Procedure

Similar to previous descriptions of vitacoxib PK in dogs (15), study protocols were reviewed and approved by the China Agricultural University Animal Care and Use Committee (Beijing, PR China). Venous blood samples were collected from pre-placed cephalic vein catheters or by venipuncture directly into 1-ml EDTA tubes. Blood samples were centrifuged at 2,280 × g for 10 min before plasma was stored at −20°C until determination of drug concentration. A 2-week washout period was scheduled between each individual study with vitacoxib. Raw data from Study 1 to Study 5 were derived from our previously published non-compartmental analysis (NCA) of vitacoxib disposition in cats (16). Further details on the experimental procedure for these studies can be found in Wang et al. (16). In brief, vitacoxib PK data were collected as follows:

- Study 1 (Single Oral Dose Fasted Conditions): Eight healthy cats received a single nominal dose of 2 mg/kg of vitacoxib P.O (Beijing Orbiepharm Co., Ltd. Beijing, PR China) after fasting for 12 h overnight.
- Study 2 (Single I.V Dose Fasted Conditions): Eight healthy cats received a single nominal dose of 2 mg/kg of vitacoxib I.V (200 mg/10 ml, Beijing Orbiepharm Co., Ltd. Beijing, PR China) via the cephalic vein following 12 h fasting overnight.
- Study 3 (Single Oral Dose Fed Conditions): Eight healthy cats received a single nominal dose of 2 mg/kg of vitacoxib P.O (Beijing Orbiepharm Co., Ltd. Beijing, PR China) 2 h after feeding.
- Study 4 and Study 5 (Dose Proportionality): Eight healthy cats received a single dose of 1 and 4 mg/kg of vitacoxib P.O (Beijing Orbiepharm Co., Ltd. Beijing, PR China) after fasting for 12 h overnight, with a 14-day washout interval in between.
- Study 6 (Steady-State Oral Pharmacokinetics): the same eight cats as in Study 1 to Study 3 were administered a 2 mg/kg oral dose of vitacoxib (Beijing Orbiepharm Co., Ltd. Beijing, PR China) for seven consecutive days under fasted conditions.

### Data Analysis

Vitacoxib concentrations in plasma samples were measured using a validated UPLC-MS/MS analytic method after precipitation of proteins by acetonitrile as previously described (21). In brief, 100 μl of plasma was mixed with methyl tert-butyl ether to precipitate plasma proteins, and the supernatant was collected. This precipitation process was carried out twice. After the second extraction, the supernatant was evaporated to dryness with nitrogen gas. Samples were later reconstituted for analysis via UPLC–MS/MS (Waters Acquity UPLC and Water Quattro Premier, Waters Co, USA). The mobile phase consisted of 0.1% formic acid (solvent A) and acetonitrile (solvent B) with a flow rate of 0.4 ml/min. The quantification and qualitative ions were m/z 347.9/269.03 and m/z 347.9/192.03 for vitacoxib and m/z 382.0/362.0 for celecoxib (internal standard).

As previously described in Wang et al. (15), the bioanalytical method used for data analysis was thoroughly validated with a lower limit of quantification (LLOQ) of 0.5 ng/ml. Calibration curves showed satisfactory linearity through a concentration range of 0.5–500 ng/ml (*R*^2^ > 0.99). Inter- and intra-day coefficients of variation were all below 10% at three increasing concentration levels (1, 20, and 200 ng/ml). The mean recoveries ranged from 94.5 to 109.7%. Protocols for data analysis and method validation complied with established guidance on bioanalytical methods development (22).

### NLME Model Building

All plasma concentration time-course data collected from the six PK studies were fitted simultaneously using non-linear mixed-effects modeling. Parameter estimation was performed using the stochastic approximation expectation maximization (SAEM) algorithm as implemented in the Monolix Suite 2019R2 (Lixoft, France). Individual model parameters were acquired *post hoc* using the mean of the full posterior distribution.

Similar to the previous description by Sheiner and Ludden (23), non-linear mixed-effects models were written as Equation (1):

$$\begin{array}{l} y_{ij}=F\left(\phi_{i},t_{ij}\right)+G\left(\phi_{i},t_{ij},\beta \right)\cdot \varepsilon_{ij},\\ j\in \{1,\ldots ,n_{i}\},\phi_{i}=\mu \cdot e^{\eta_{i}},\text{i}=1,\ldots ,\text{N} \end{array}$$

where ***y***_***ij***_ is the observed value (i.e., vitacoxib concentration) for individual ***i*** at time ***t***_***ij***_. ***F***(****ϕ****_***i***_**, *t***_***ij***_) is the individual prediction for individual **i**, with parameter vector ****ϕ****_***i***_, at time ***t***_***ij***_. ****ε****_***ij***_ is an independent random variable. The function ***G***(****ϕ****_***i***_**, *t***_***ij***_**, **β****) determines the scale of the random error for a given individual **i**, at a given time ***t***_***ij***_, with covariates ****β****.

As described in Mochel et al. (24), ***F***(****ϕ****_***i***_**, *t***_***ij***_) refers to the structural model, while ***G***(****ϕ****_***i***_**, *t***_***ij***_**, **β****) is known as the residual error model (a combination of unexplained variability and measurement noise). ****μ**** represents the typical value (can be approximated by the population average) of a model parameter. Additionally, the sources of variation between the individual parameters ****ϕ****_***i***_ can be explained by both covariates and inter-individual variability (IIV). The independent random variables ****η****_**i**_ represent the IIV between parameters ****ϕ****_***i***_ and the population average ****μ****.

The random variables ****ε****_***ij***_ and ****η****_***i***_ were assumed to be normally distributed with mean value 0 and variance–covariance matrices ****σ****^**2**^ and ****ω****^**2**^**, ** respectively. Consequently, the individual parameters ****ϕ****_***i***_ are log-normally distributed.

### Model Evaluation

Convergence of the SAEM algorithm was assessed by inspection of both the stability of the fixed and random effect parameter search, as well as the stability of the log-likelihood estimate after the exploratory period of the algorithm (i.e., after 1,000 iterations of the SAEM). Standard goodness-of-fit (GOF) plots, including individual predictions vs. observations, the distributions of weighted residuals (IWRES), and normalized prediction distribution errors (NPDE), were used to assess the performance of the candidate models. For GOF diagnostics, a suitable model should have the following features: (*i*) the line of identity is aligned with the regression line (for both individual and population predictions), while (*ii*) the model residues are centered on a mean value of 0, with (*iii*) a homogeneous dispersion around the mean (24, 25). Similar to our previous description of vitacoxib PK in dogs (15), prediction distributions derived from 500 Monte Carlo simulations were used to evaluate the ability of the final model to reproduce the variability in the observed PK data. Likewise, and as described in Wang et al. (15), “residual error estimates from the mathematical models were used as supportive information for evaluation of goodness of fit. Normality and independence of residuals were assessed using histograms, quantile–quantile plots, and autocorrelation of conditional weighted residuals. For converging models with satisfactory goodness-of-fit diagnostics, model selection was based on the Bayesian information criteria (BIC) and the precision of the model parameter estimates. The BIC was selected over the Akaike Information Criterion as it tends to select simpler and more parsimonious models” (26).

All IIV and inter-occasion variability (IOV) terms were modeled using log-normal distributions except for parameters of the oral absorption function, which were modeled using a logit-normal function to bound predictions between 0 and 1.

### Handling of Below Limit of Quantification (BLQ) Data

Data below the lower limit of quantification (LLOQ) were modeled by adding to the likelihood function a term describing the probability that the true observation lies between zero and the LLOQ, which is equivalent to the M3 method implemented in the NONMEM (Non-linear Mixed Effects Modeling) software.

### Parameter Correlation Estimates

Visual inspection of the eta vs. eta scatterplots as well as results from the Pearson's correlation tests were used to inform our choice of correlations between model parameters. In agreement with previous literature (17, 27), multiple samples from the posterior distribution obtained at the last iteration of the SAEM were used during the evaluation of parameter correlations. Final inclusion of correlations in the structural model was determined by changes in the BIC value as well as precision of parameter estimates.

### Inclusion of Covariate Relationships

The significance of bodyweight, sex, and feeding status on parameters estimates was evaluated using the automated Pearson's correlation test and ANOVA method as implemented in Monolix 2019R2. As well as evaluating bodyweight as a continuous covariate, we evaluated log-normalized bodyweight during the covariate search, i.e., $log\;normalized\;BW=log\;\left(\frac{bodyweight}{weighted\;mean\;bodyweight}\right)$. If a covariate met the threshold of *P* < 0.05 (Pearson's test for continuous covariates and ANOVA for categorical), it was evaluated for inclusion in the model. Inclusion of covariates in the final model was determined by BIC as well as precision of final parameter estimates.

### Whole Blood Assays to Derive Pharmacodynamic Targets

The potency and selectivity of vitacoxib were determined in whole blood assays from the same species, as previously described by Giraudel et al. (28). Blood samples were collected from seven healthy, neutered, domestic short-hair cats. Coagulation-induced thromboxane and lipopolysaccharide-induced prostaglandin E2 concentrations were used to determine the selectivity of vitacoxib for the COX-1 and COX-2 enzymes, respectively. Assays were performed using whole blood with and without the addition of vitacoxib at increasing levels. For COX-1, 1 ml of blood was collected from each cat into anticoagulant free tubes. Then, aliquots of blood (199 μl) were mixed with 1 μl of dimethylsulfoxide (DMSO) containing vitacoxib to reach a final concentration ranging from 0.03 to 500 μM. Blood aliquots containing only DMSO were used as controls. The tubes were vortexed (10 s) and then incubated for 1 h at 37°C. After centrifuging at 2,000 × g (4°C for 10 min), the supernatant was collected and stored at −80°C before being analyzed for TXB2 using commercial ELISA kits (TXB2 ELISA kit-480 wells, Cayman). For COX-2, 1 ml of blood was collected from each cat into heparinized tubes. Aliquots (199 μl) of blood were added to microtubes containing a range of concentrations of vitacoxib dissolved in 1 μl of DMSO to reach a final concentration ranging from 0.0019 to 31.25 μM. Then, lipopolysaccharide (LPS) [5 μg/ml in sterile phosphate buffered saline (PBS)] was added and vortexed. For each cat, two aliquots were incubated with or without LPS to obtain a positive and negative control, respectively. All samples were incubated for 24 h at 37°C. The supernatant was collected and stored at −80°C prior to analysis for PGE2 using an ELISA assay kit (PGE2 ELISA kit-480 wells, Cayman).

### Monte Carlo Simulations

After final model selection and fit, Monte Carlo simulations were used to predict the expected time course of potential vitacoxib dosing schedules. To do this, the average plasma time course of vitacoxib was simulated using the parameter estimates from the final model (with IIV and model error fixed to zero) from 0.01 to 8 mg/kg input doses, after either I.V or P.O administration, and under fed or fasted conditions.

Simulations were then used to derive the average amount of time vitacoxib plasma concentrations remained above one of four pharmacodynamic targets: IC_10_ and IC_20_ against COX-1, and IC_80_ and IC_90_ against COX-2, as metrics of safety and efficacy, respectively. Coxib dosages that result in more than 80–90% of COX-2 inhibition and <10–20% COX-1 inhibition over most of the dosing interval are likely to be relatively safe in terms of gastrointestinal and platelet side effects (28).

Simulations were performed in R 3.6.2 (The R Foundation for Statistical Computing), using the mlxR package (maintained by Lixoft, France).

## Results

### Animal Safety

No adverse drug effects were reported after vitacoxib dosing in cats in any of the six PK studies.

### PK Model Evaluation

**Figure 1:**
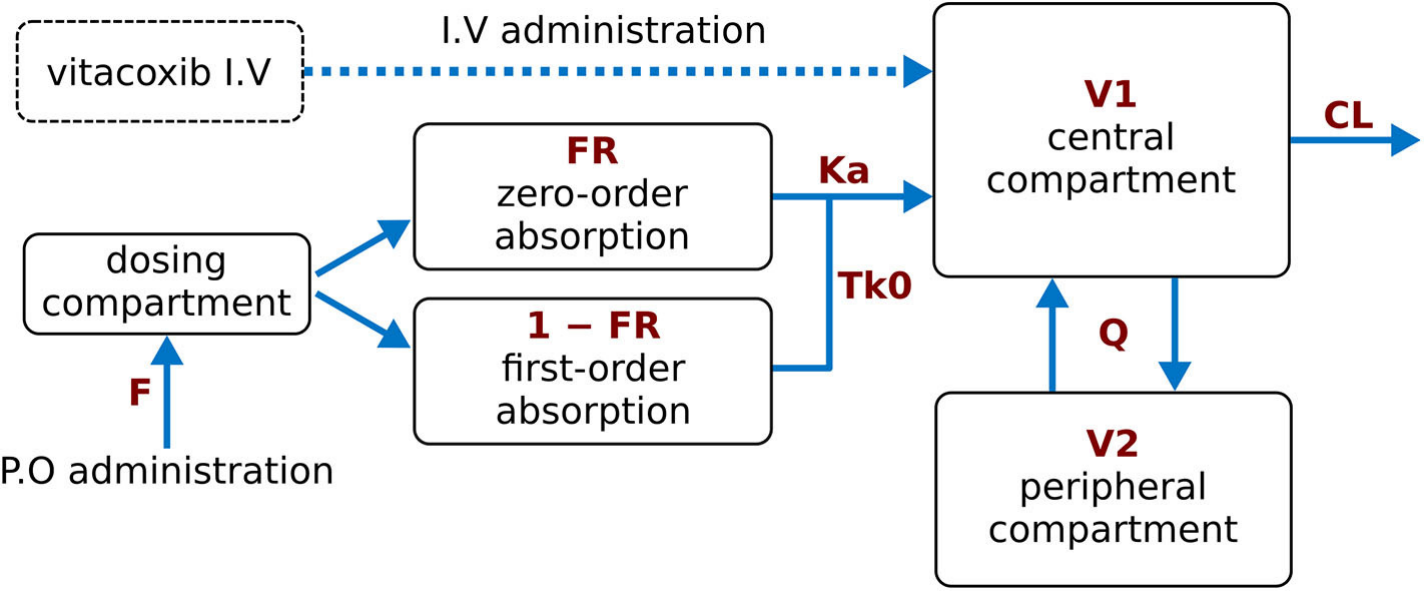
Final model structure of vitacoxib pharmacokinetics following I.V and P.O dosing in healthy cats. A two-compartment pharmacokinetic model, with mixed first-order and zero-order absorption for the P.O route, best described the pharmacokinetics of vitacoxib in plasma. CL: vitacoxib systemic clearance; Q: intercompartmental clearance; V1: central volume of distribution; V2: peripheral volume of distribution.

A two-compartment mammillary disposition model—including simultaneous first- and zero-order absorption for the P.O. route—(17) best described the PK of vitacoxib in plasma (Figure 1). In the absorption model, the first-order absorption rate was represented by **ka**, and the zero-order absorption rate constant was represented by **Tk0**. The fraction of drug absorbed by the first- and zero-order rate constant was represented by **Fr** and (**1 – Fr**), respectively.

**Figure 2:**
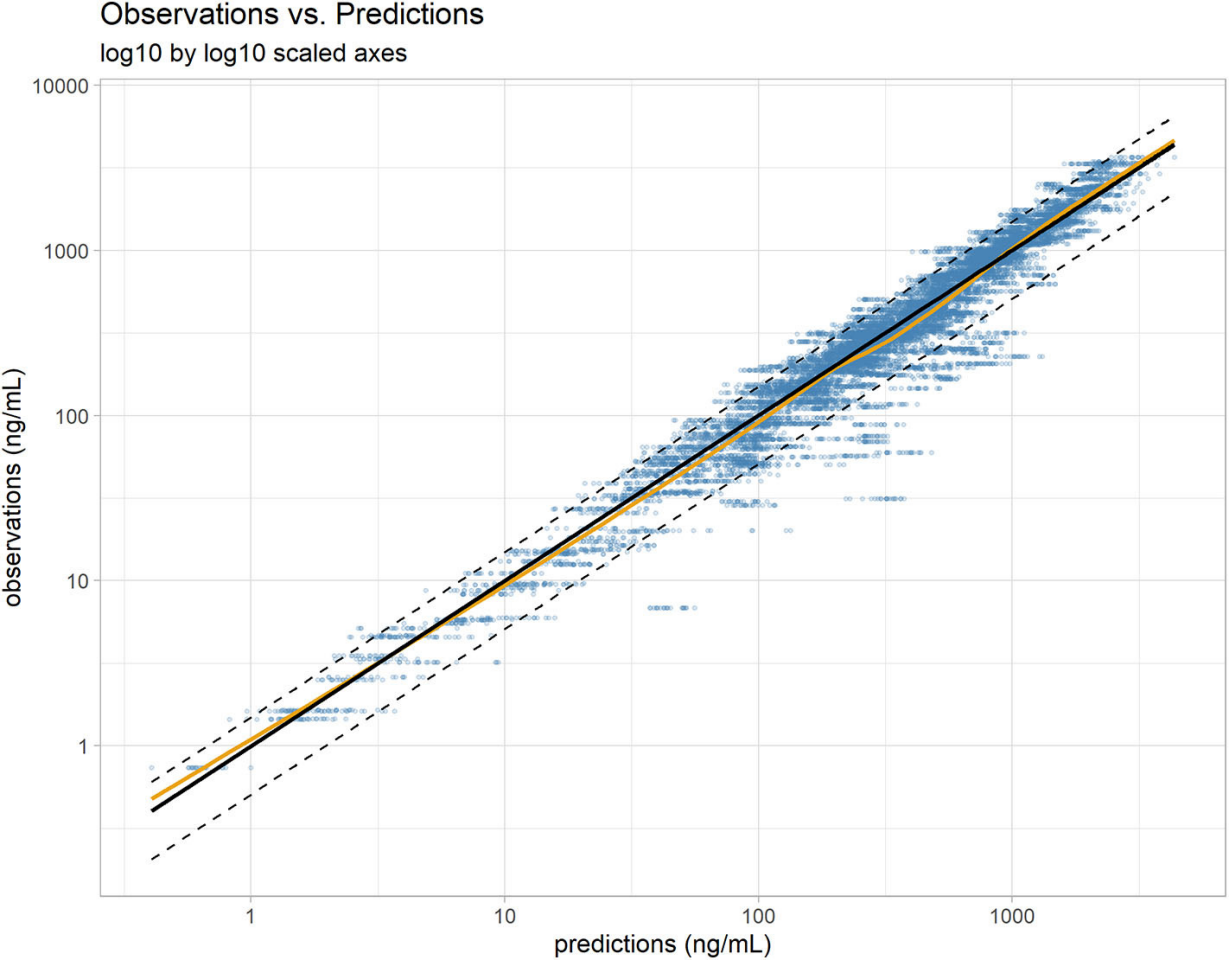
Individual predictions vs. observations. The robustness of fit and predictive performances of the final model were supported by the relatively consistent agreement between observations and individual predictions. Blue dots: observations; black line: identity line; dotted black lines: 90% prediction interval; yellow line: spline. Plotted with log10 by log10 scaled axis to improve ability to compare observations across a large range of values.

Similar to Wang et al. (15), a proportional error model was used to account for the residual error in the measurement of vitacoxib in plasma. The robustness and predictive performance of the final model was supported by several standard goodness-of-fit diagnostic plots such as observations vs. predictions (Figure 2) and the model residuals (Supplementary Figure A). The normality of the random effects was further supported by the distribution of the η_*i*_ around a mean value of 0 (Supplementary Figure B).

**Figure 3:**
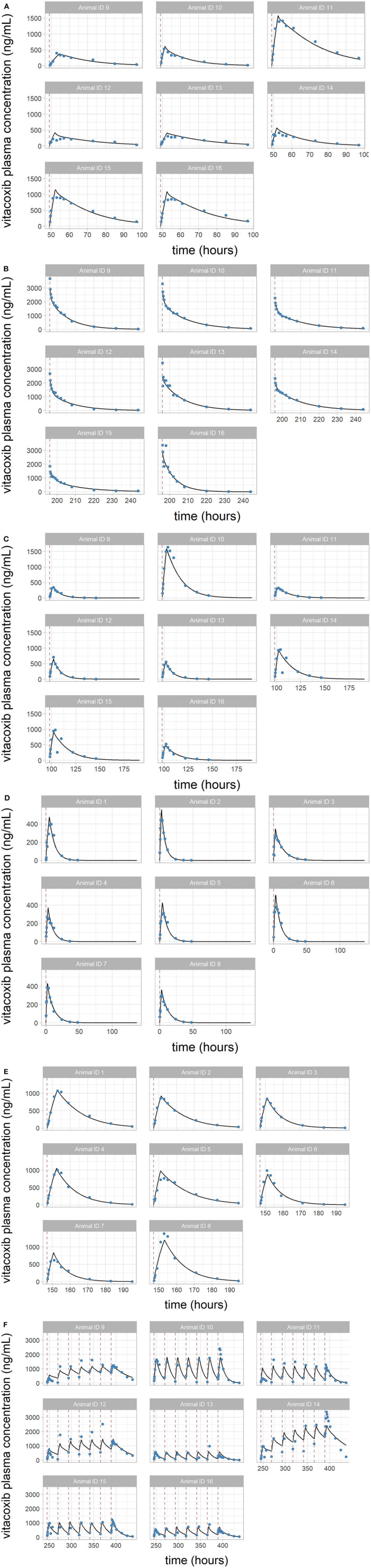
**(A–F)** Individual predictions of vitacoxib plasma concentration in cats from the final selected model. Plots of individual observed (blue dot) and individual predicted (black line) vitacoxib concentration time course. Dosing events are indicated by dotted red lines. The full model was able to describe the individual time course of vitacoxib for all dosing schedules with excellent accuracy, as shown by the quality of the individual fits.

**Figure 4:**
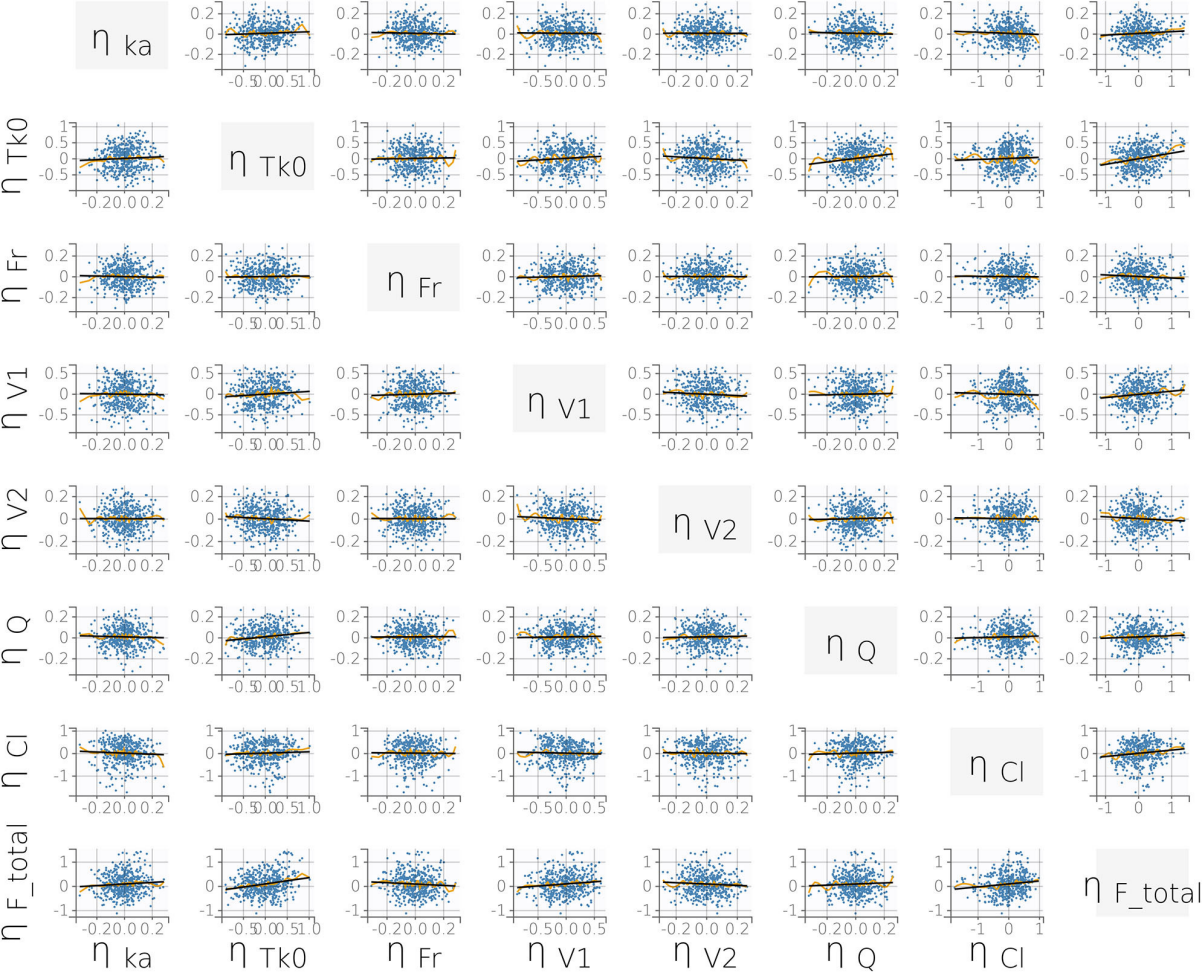
Correlation plot matrix of the random effects (i.e., the η_*i*_). All correlation coefficients were estimated to be low (*r* ≤ 0.15) and did not meet the threshold for inclusion (Pearson's correlation test, *P* < 0.05). This supports our final statistical model choice. Yellow lines are splines of correlation while black lines are simple linear regressions.

Overall, the model was able to reproduce the individual variability in vitacoxib concentration kinetics with little individual error (Figures 3A–F). Results from the automated covariate search, as implemented in Monolix 2019R2, identified bodyweight as a significant covariate on vitacoxib central compartment volume (Equation 2).

$$\begin{array}{l} log\left({V_{1}}_{i}\right)=log\left(V_{1pop}\right)+\beta_{V_{1}WT0}\cdot {WT0}_{i}+{\eta_{V1}}_{i} \end{array}$$

where *V*_1*pop*_ is the population central compartment volume of distribution, β_*V*_1_*WT*0_ is the effect of the continuous covariate (bodyweight) on *V*_1_, WT0_i_ is the individual weight, and _η_*V*1_*i*_ is the individual random effect. No significant statistical correlations between parameters were found during the model building process (Figure 4).

**Figure 5:**
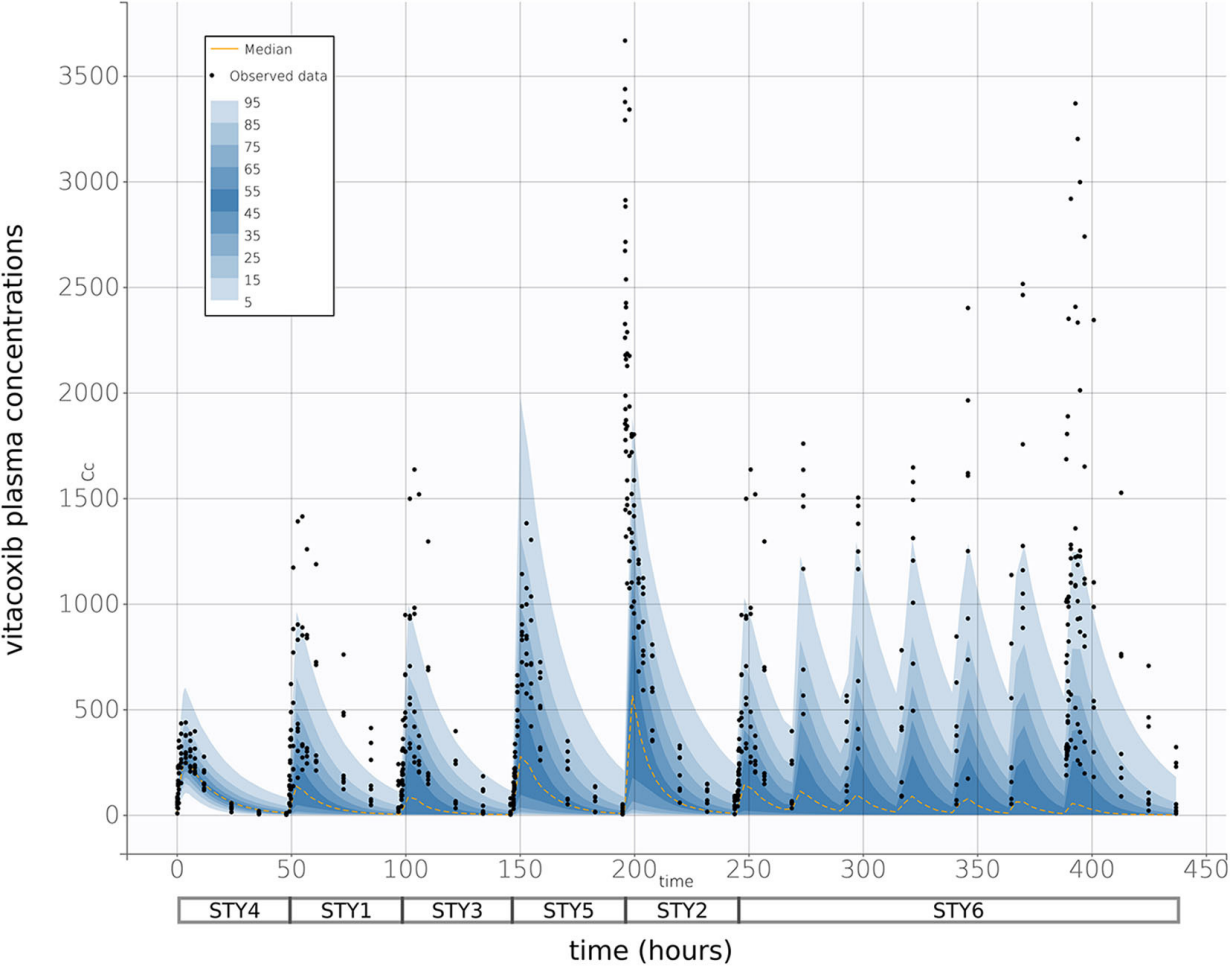
Prediction distribution vs. observations. As described in Wang et al. (15), theoretical distribution of predictions was produced by 500 Monte Carlo simulation from the model fit. Briefly, the experiment was replicated virtually 500 times, allowing for each quantile (from 5 to 95 in steps of 5, i.e., {5, 10, 15,…,90, 95}) to be estimated 500 times. The blue areas are ranges of quantiles and the black points are observations for comparison. The yellow lines are median predictions, and studies are aligned sequentially—as indicated by the boxes below the *x*-axis. With a 95% prediction interval, we would expect that there would be some degree of misspecification, but the high general correspondence between model and observations indicate a strong fit performance.

Finally, prediction distributions derived from 500 Monte Carlo simulations further supported the predictive quality of the final selected model, which was able to accurately reproduce the variability in the PK of vitacoxib in cats after I.V and P.O (single and multiple) dosing (Figure 5).

### Parameter Estimates

Final parameter estimates are summarized in Table 2. Precision of the final parameter estimates was overall very satisfactory (i.e., RSE ≤ ~25% for all PK parameters). The total systemic clearance of vitacoxib was estimated at 110 ml/h, with a total VSS of 3.42 L; the central compartment occupying the majority of the volume of distribution of vitacoxib in cats. Lastly, the oral bioavailability of vitacoxib in cats was estimated to be moderate to high (~60%).

**Table 2:** Estimated model parameters and their associated relative standard errors (RSE%) and variation (CV%) for vitacoxib pharmacokinetics in cats.

**Parameter**	**Symbol**	**Unit**	**Point estimate**	**Relative Standard Error (%)**	**CV (%)**
First-order absorption rate constant (P.O)	Ka	1/h	0.13	15.4	10.0
Zero-order absorption rate constant (P.O)	Tk0	h	3.76	6.4	31.5
Fraction absorbed through 1st order	Fr	%	0.20	16.4	8.0
Central compartment volume of distribution	V1	L	2.88	25.1	37.4
Peripheral compartment volume of distribution	V2	L	0.54	19.4	109
Inter-compartmental clearance	Q	L/h	0.52	6.7	10.0
Systemic Clearance	CL	L/h	0.11	7.9	46.0
Bioavailability (P.O)	F	%	57.8	7.1	26.5
Bodyweight effect on V1	β_*V*1_*WT*0_	L	0.41	20.1	-
Proportional error constant	b	-	0.30	3.1	-

*Random effects are expressed in terms of IOV (expressed as CV%) given the nature of the experimental design. -: not applicable. Bodyweight had a significant effect on the volume of the central compartment (P < 0.001). Additional details on the definition of the abbreviated parameters can be found in the caption of Figure 1*.

For model building, the statistical model was first parameterized using a full matrix of IIV and IOV random effects. However, when estimating the full variance–covariance matrix, several IIV and IOV random effects converged toward zero (<1e−4) and were estimated with low precision (i.e., high RSE%). Therefore, these low-precision and zero-convergent random effects were set to 0.1 (CV ~10%), as is standard practice in model building. Most of the variance in the estimated model parameters originated from IOV (i.e., within-subject). Vitacoxib systemic clearance and the volume of distribution of the central compartment drove the majority of the variability in the model (Table 2).

Bodyweight had a significant effect on the volume of distribution for the central compartment (*P* < 0.001) (Table 2). However, results from the automated covariate analysis in Monolix 2019R2 suggested that neither age, feeding status, nor sex had a statistically significant effect on the PK of vitacoxib in cats.

### Model Simulations

Results from whole blood assays with vitacoxib provided the following estimates for inhibition of the cyclooxygenase isoenzymes:

- COX-1 IC_10_: 911.3 ng/ml
- COX-1 IC_20_: 1467.8 ng/ml
- COX-2 IC_80_: 313.0 ng/ml
- COX-2 IC_90_: 556.5 ng/ml

**Figure 6:**
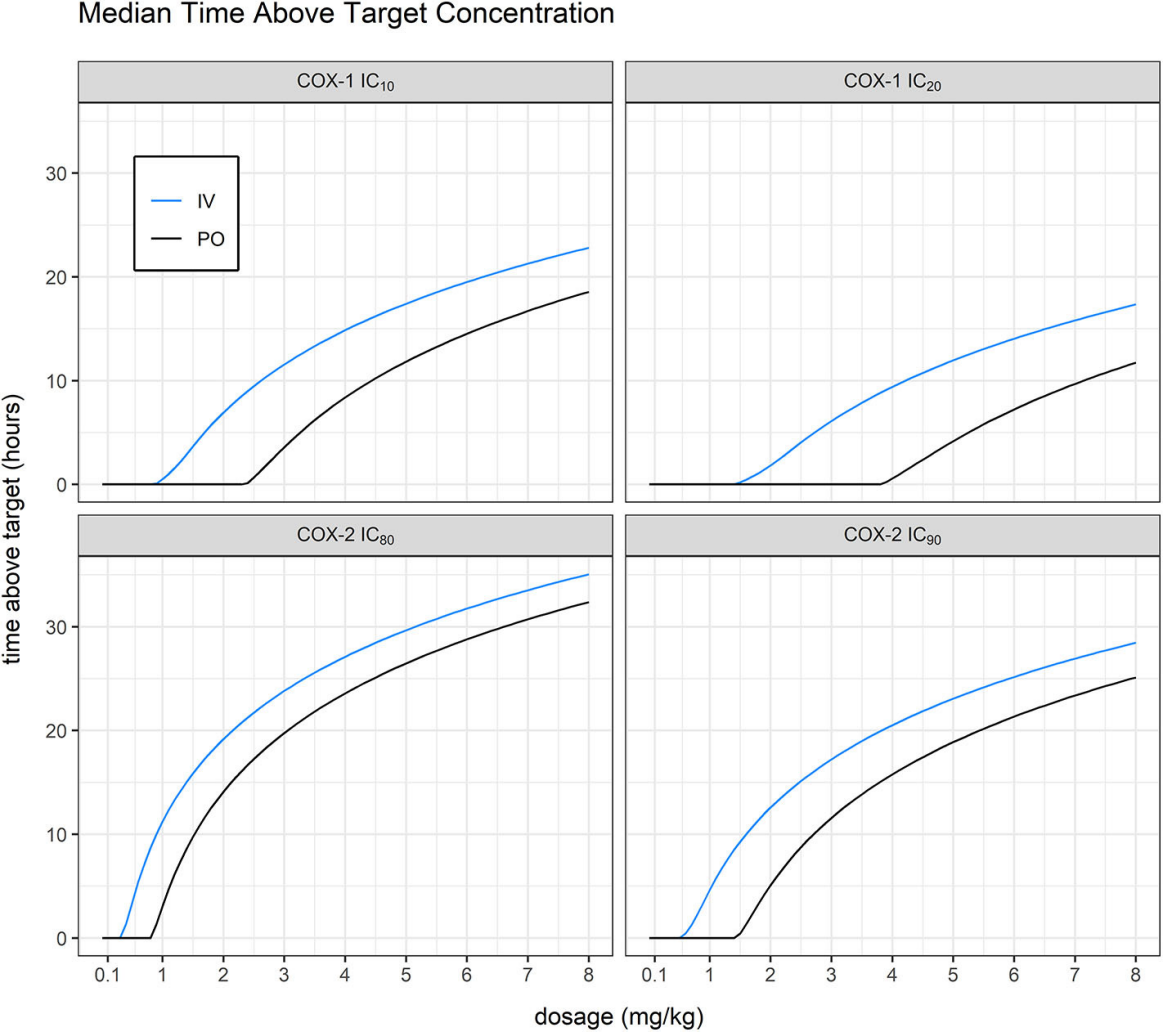
Mean time above target vs. dosage. Simulating without inter-occasion/individual variability, we were able to derive average predicted time above target for IC_10_ and IC_20_ against COX-1 (911.28 and 1467.8 ng/ml, in-house unpublished data) and IC_80_ and IC_90_ against COX-2 (313.03 and 556.512 ng/ml, in-house unpublished data). Model-derived simulations showed that an oral dose of vitacoxib at 2 mg/kg produced systemic concentrations above the IC_80_ of COX-2 for ~12 h, with no effect on COX-1.

As expected, we found that the dosing route had a significant effect on total time above target plasma concentration for vitacoxib in cats (Figure 6). In all cases, our simulations confirmed that vitacoxib was a highly selective COX-2 inhibitor. In fact, our model-derived simulations showed that an oral dose of vitacoxib at 2 mg/kg produced systemic concentrations above the IC_80_ of COX-2 for ~12 h, with no effect on COX-1. Additionally, an oral dose of 4 mg/kg would maintain systemic concentrations of vitacoxib above the IC_80_ of COX-2 for about 24 h, with modest to minimal effect on COX-1 based on available IC_20_ and IC_10_ estimates.

## Discussion

In the present study, PK data resulting from I.V and P.O administration of single and multiple doses of vitacoxib, in cats, were modeled using non-linear mixed-effects. To the best of our knowledge, this is the first comprehensive attempt to model vitacoxib disposition kinetics, in cats, from various experimental settings. Compared with previous descriptions of vitacoxib PK in cats (16), the current analysis provides a mathematical representation of vitacoxib disposition that allowed simulations of its concentration time course for doses that have not been tested during the original study design. This is a significant departure from Wang et al. (16) as we were later able to correlate these simulated concentrations to the expected efficacy of the NSAID to predict a range of therapeutic doses in cats. Additionally, our mathematical model allowed us to investigate the effect of population characteristics such as bodyweight, sex, or feeding status on the drug PK, which should, potentially, be taken into for dose selection.

A two-compartment mammillary disposition model, with simultaneous first- and zero-order absorption for the P.O route, best described the PK of vitacoxib in plasma. A similar model has been used to describe the disposition of another coxib, robenacoxib, in cats (17). However, the model used in this study to describe vitacoxib PK, in cats, slightly differs from previously reported vitacoxib PK modeling efforts, in dogs (15). In previous efforts, a single first-order absorption function was used for the modeling of oral absorption in canines. Parameter estimates from the final model suggest that the systemic clearance of vitacoxib is low (110 ml/h equivalent to 1.8 ml/min), consistent with a small extraction ratio (E < 0.01). This could be related to reduced glucuronidation capacity in cats (3).

The volume of distribution of vitacoxib in cats was estimated to be relatively small (3.42 L, i.e., ~1.18 L/kg), which is typical for NSAIDs that are highly bound to plasma proteins such as albumin, and limits their distribution to the extracellular space (29). Of note, total bodyweight had a statistically significant effect on vitacoxib central volume of distribution. The moderate distribution volume of vitacoxib together with its low systemic clearance results in an estimated half-life of ~21 h in cats (0.693 × VSS/Cl), which is greater than previously reported values in dogs (~12.7 h) (14). Further ADME studies are warranted to better characterize the tissue distribution of vitacoxib in cats. Because we are using a somewhat empirical model of vitacoxib PK, we cannot determine the exact nature of the mechanisms contributing to the first pass of vitacoxib in cats. However, we do hypothesize that a significant fraction of vitacoxib does not cross the intestinal barrier and is not available systematically. This pre-systemic intestinal loss would support our observation of a moderate oral bioavailability (~60%) besides a low extraction ratio.

Results from the covariate analysis on this preliminary dataset do not suggest any impact of sex, bodyweight, and food intake on vitacoxib clearance in cats, therefore limiting the need for dosage adjustment based on these covariates in this species. However, these findings need to be verified in a larger dataset including a more diverse population of cats.

Of note, the variance in individual parameters was primarily driven by IOV (i.e., within-subject), rather than IIV, primarily due to the nature of the experimental design (using the same eight cats in four out of six studies and then another set of eight cats in Study 4 and Study 5).

Finally, using NLME PK modeling rather than an NCA allowed us to make meaningful predictions of the effects of vitacoxib under multiple dosing (“what if”) scenarios (30). As previously documented, cats are especially sensitive to the side effects of coxibs due to their low capacity for glucuronidation. This sensitivity, along with financial constraints, limits the practicality of performing a suite of dosage experiments to empirically determine the optimal dosage of vitacoxib in this species. In this paper, we have provided a modeling and simulation framework to guide future experiments in optimizing vitacoxib dosage in cats. Using this framework, we have made a first estimate of a meaningful range of optimized vitacoxib dosages for further testing in clinical trials, in actual cat patients.

### Limitations

In this study, neither age or breed differences could be accounted for during model building. Importantly, our findings on vitacoxib PK in cats were derived from a small sample of healthy individuals, which somewhat hampered our ability to assess the effect of individual covariates on vitacoxib PK in cats. Our preliminary results therefore need to be confirmed in a larger study population where the effect of breeds, age, sex, and disease can be further evaluated.

Additionally, clinical pathology (biochemistry and hematology) parameters were not monitored during the course of these pilot PK studies because a more thorough evaluation of vitacoxib safety in cats is required prior to subsequent clinical testing in client-owned animals. An additional limitation is the fact that we did not measure COX inhibition directly in the blood and synovial fluid in this study, such that data used for simulations of times above different ICs for COX inhibition were derived from *in vitro* whole blood assays.

## Conclusion

In summary, a two-compartment mammillary disposition model, with simultaneous first- and zero-order absorption for the P.O. route, best described the PK of vitacoxib in cats. Vitacoxib, a poorly soluble and highly permeating NSAID, has a low systemic clearance and a limited volume of distribution in cats. Our model provides a simulation framework to optimize vitacoxib dosing strategies in cats prior to *in vivo* experiments in feline patients.

## Data Availability Statement

The data analyzed in this study is subject to the following licenses/restrictions: Sponsored research - the data are proprietary. Requests to access these datasets should be directed to Beijing Orbiepharm Co. Ltd., Beijing, China.

## Ethics Statement

The animal study was reviewed and approved by China Agricultural University Animal Care and Use Committee. The study exclusively used experimental animals.

## Author Contributions

JW and XC contributed to the design and execution of the experiments. JM and BS performed the NLME data analysis and wrote the manuscript together with JW. JL provided the test drug. XG and JQ contributed to the animal experiments. All authors have read and approved the final manuscript.

## Conflict of Interest

JL was employed Beijing Orbiepharm Co. Ltd. The remaining authors declare that the research was conducted in the absence of any commercial or financial relationships that could be construed as a potential conflict of interest.
