# Supplementary material for: Non-Linear Mixed-Effects Pharmacokinetic Modeling of the Novel COX-2 Selective Inhibitor Vitacoxib in Cats
Source: Front Vet Sci. 2020 Sep 24;7:554033. doi: 10.3389/fvets.2020.554033 (PMC7546276; doi:10.3389/fvets.2020.554033)
Supplement: Supplementary file 1 [file Data_Sheet_1.pdf]

Supplementary Material

Supplemental Figure (A): Normalized Prediction Distribution Error

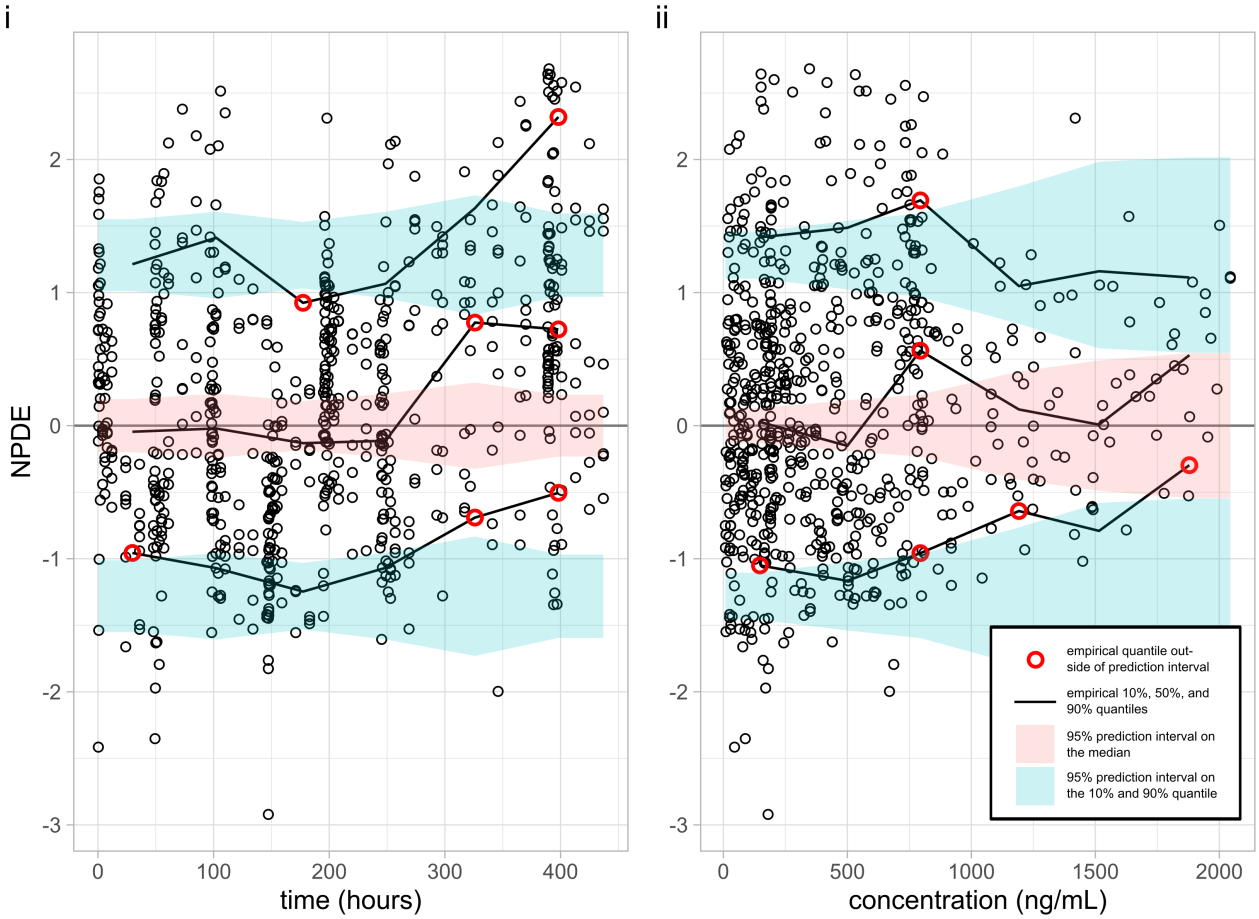

<u>Supplemental Figure (A).</u> Normalized prediction distribution errors:
Normalized prediction distribution error (NPDE) vs. time (left panel, [i]) and concentration (right panel, [ii]). The black lines are the empirical 10%, 50%, and 90% quantiles. The blue and red ribbons are the 95% prediction intervals about each respective empirical quantile. Red circles indicate where the empirical quantile falls outside of the prediction interval and can be an indication of misspecification. As described by Comets et al. (1), prediction distribution errors (PDE) are obtained after decorrelation of the observed and simulated concentrations with respect to the empirical mean and variance obtained in the simulations. PDE are then normalized by using the inverse of the cumulative density function of a normal distribution of mean 0 and variance 1. Other than a slight misspecification for large concentrations and in the tail region of the multidose regimen, the empirical quantiles fall within the

prediction intervals and the NPDEs are evenly distributed about zero – especially in the regions where sampling density was highest. This indicates that our model can explain the majority of variation in the data. Simulations and plotting were done in R version 4.0.2, using the packages mlxR 4.1.2, ggplot 2 3.3.2, and patchwork 1.0.1.

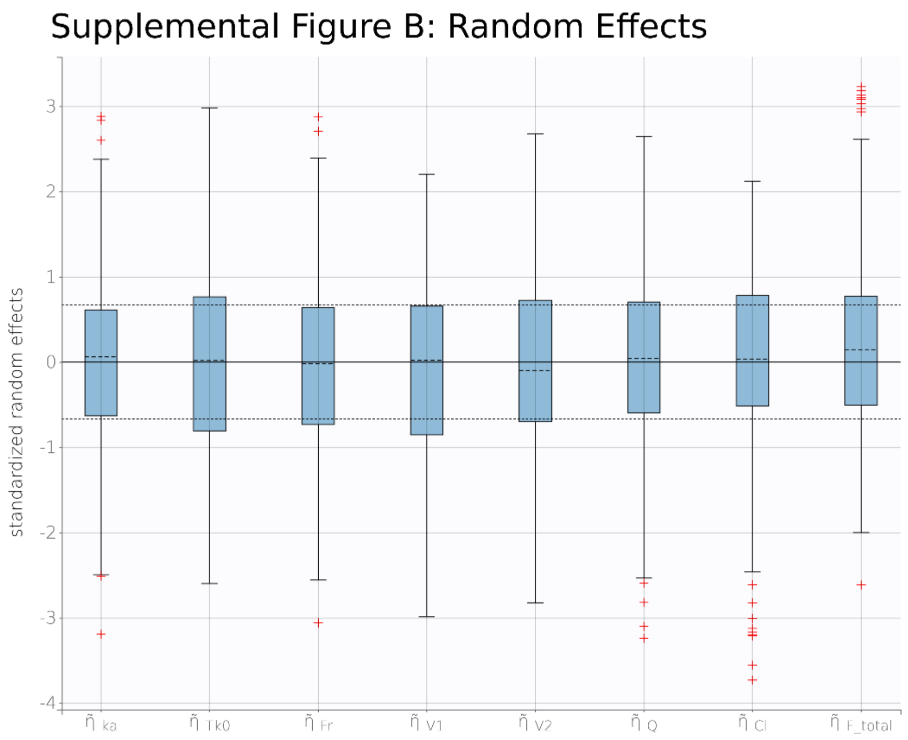

<u>**Supplemental Figure (B).**</u> **Probability distribution of the random effects (η_i):**

Dotted line: theoretical interquartile range; blue area: empirical interquartile range. The normality of the random effects was supported by the homogeneous distribution of the random effects (η_i) around a mean value of 0.

REFERENCES

1. Comets E, Brendel K, Mentré F. Computing normalised prediction distribution errors to evaluate nonlinear mixed-effect models: the npde add-on package for R. *Comput Methods Prog Biomed*. (2008) 90:154–66. doi: 10.1016/j.cmpb.2007.12.002
